# Advanced distributed feedback lasers based on composite fiber heavily doped with erbium ions

**DOI:** 10.1038/s41598-020-71432-w

**Published:** 2020-09-02

**Authors:** Mikhail I. Skvortsov, Alexey A. Wolf, Alexander A. Vlasov, Kseniya V. Proskurina, Alexander V. Dostovalov, Olga N. Egorova, Boris I. Galagan, Sergey E. Sverchkov, Boris I. Denker, Sergey L. Semjonov, Sergey A. Babin

**Affiliations:** 1 Institute of Automation and Electrometry of the Siberian Branch, Russian Academy of Sciences, Novosibirsk, Russia; 2 Prokhorov General Physics Institute of the Russian Academy of Sciences, Russian Academy of Sciences, Moscow, Russia; 3 Dianov Fiber Optics Research Center, Prokhorov General Physics Institute of the Russian Academy of Sciences, Moscow, Russia

**Keywords:** Fibre lasers, Fibre optics and optical communications, Laser material processing

## Abstract

Specially designed composite heavily Er^3+^-doped fiber in combination with unique point-by-point inscription technology by femtosecond pulses at 1,026 nm enables formation of distributed-feedback (DFB) laser with ultra-short cavity length of 5.3 mm whose parameters are comparable and even better than those for conventional Er^3+^-doped fiber DFB lasers having much longer cavity. The composite fiber was fabricated by melting rare-earth doped phosphate glass in silica tube. The ultra-short DFB laser generates single-polarization single-frequency radiation at 1,550 nm with narrow linewidth (3.5 kHz) and 0.5 mW output power at 600 mW 980-nm pumping. The same fiber with conventional CW UV (244 nm) inscription technology using phase mask enables fabrication of 40-mm long DFB laser with > 18 mW output power at 3.3% pump conversion, which is a record efficiency for Er^3+^-doped fiber DFB lasers. The developed technologies form an advanced platform for Er^3+^-doped fiber DFB lasers operating around 1.55 µm with excellent output characteristics and unique practical features, in particular, the ultra-short DFB lasers are attractive for sensing applications.

## Introduction

Distributed feedback (DFB) fiber lasers are known as a versatile source of single-frequency radiation for a wide variety of applications from high resolution spectroscopy^1^ to precision sensing^2,3^, thanks to the high stability, low noise level (SNR ~ 70 dB), and narrow linewidth (~ 1 kHz). A DFB fiber laser cavity is formed by a single π-phase-shifted fiber Bragg grating (FBG) inscribed directly in an active fiber. Such integrated structure is usually more stable than the cavity of distributed Bragg reflector (DBR) fiber laser consisting of two passive FBGs with a piece of active fiber between them.

Typical length of DFB and DBR fiber laser cavity is usually amounts to several centimeters that may be too much for some specific applications where they are used as active point-action sensors^3^ which are much more sensitive and precise than conventional passive FBG sensors. Moreover, they may be easily assembled into an array of sensors providing new capabilities such as three-element vector hydrophone with high sensitivity^4^, which has extensive applications to underwater acoustic monitoring for defense, oil & gas exploration etc. For such applications, fiber lasers with cavity length of ≤ 1 cm are in a great demand. In addition, such short DFB lasers are attractive from the point of their fabrication (reduced errors) and packaging (compact footprint and lower sensitivity to environment).

Development in this direction resulted in demonstration of Er/Yb co-doped fiber lasers with 8.4-mm long DBR cavity providing single-frequency generation near 1.54 µm with ≈ 3 kHz linewidth^5^. Taking in mind that the DBR lasers have significant drawbacks such as mode hopping, it is more attractive to develop short DFB lasers. As known from the literature, the shortest DFB fiber lasers have 16-mm long cavity in Yb^3+^-doped fiber^6^ and 17–20 mm cavity in Er^3+^-doped fiber^7,8^ reasoned mainly by the absence of active fibers with high gain at low concentration of clusters (ion pairs) limiting CW single-frequency regime of generation in DFB lasers. The highest level of the fiber core doping by rare-earth ions at the minimum level of clustering in case of silica fibers has been obtained by implementing the preform’s manufacturing technology of surface plasma chemical vapor deposition of silica glass at reduced pressure (SPCVD)^6,8,9^ featured by the absence of the glass-melting stage during the synthesis process.

Another possibility is to explore FBG inscription in phosphate fibers enabling even higher doping concentration at weaker cluster formation^10^. However, it is difficult to directly inscribe a short in-core Bragg grating using traditional inscription technologies, due to the sufficiently lower photosensitivity of phosphate fibers as compared with silica fibers. In Er/Yb co-doped phosphate fibers, it is shown to be possible to obtain an efficient DFB lasing at direct UV inscription of a ~ 5 cm long phase-shifted FBG, see^11^ and citation therein.

In this paper, we fabricate DFB laser in a composite fiber with high Er^3+^ concentration (3 wt%). The composite fiber was fabricated by melting rare-earth doped phosphate glass in silica tube^12,13^. Since the presence of phosphorus oxide reduces photosensitivity, femtosecond technology enabling FBG fabrication in non-photosensitive fibers, has been applied. As a result, a CW DFB laser operating at 1,550 nm with record cavity length of 5.3 mm has been demonstrated. For the sake of comparison, we have also performed test fabrication of DFB laser in the composite fiber by traditional CW UV inscription technology with a phase mask^14^. Due to the lack of photosensitivity, it is hardly possible to fabricate by the UV technology a short laser, but 40-mm long DFB laser has been developed exhibiting unique characteristics, one of which is record efficiency (3.3%) for Er^3+^-doped fibers.

## Experiments and results

### Active fiber characterization

The optical fiber used as an active medium for the DFB lasers was made by sintering phosphate glass in a silica tube and then drawing the preform. The process of manufacturing a fiber is described in detail in^12,15^. For the core manufacturing we used glass of the same composition as in the works^12,15^. In addition to 65 mol% of phosphorus oxide (PO_2_), this composition contained 7 mol% of Al_2_O_3_, 12 mol% of B_2_O_3_, 9 mol% of Li_2_O, and 7 mol% of RE_2_O_3_^16^. The concentration of erbium oxide in the initial glass was about 1.2 mol% (3 wt% erbium). This composition also contained gadolinium; the total concentration of rare earth oxides was about 7 mol%. As noted in the works^12,15^, when a preform with a core consisting of a phosphate glass and a cladding consisting of silica is drawn into a fiber, there is a significant mutual diffusion between these glasses. To determine the degree of mutual diffusion in the fiber, the concentration of phosphorus oxide in the core was estimated by energy dispersive analysis. According to the estimate, the concentration of phosphorus oxide was about 26 mol%. To assess the concentration of aluminum, lithium, boron, and gadolinium oxides that make up the initial glass, we can assume that their concentration decreases in proportion to the decrease in the concentration of phosphorus oxide in the core of the fiber compared to the original glass.

The core diameter of the fabricated fiber, measured from the end face image obtained using an electron microscope, is about 4.0 μm. The diameter of the quartz glass cladding is 100 μm. Near the wavelength of 1.55 μm we have also studied the mode composition of the fabricated fiber—under various excitation conditions at the input end, only the intensity distribution corresponding to the fundamental mode of the fiber core is observed at the output end of the fiber. This indicates that the fabricated fiber operates in single-mode regime near 1.55 μm. Using a section of a fiber with a length of 2 m, the cut-off wavelength of the first higher mode has been measured using the standard bending method. The measured value is 1.3 μm. The mode field diameter at 1.55 μm was 4.5 μm.

**Figure 1:**
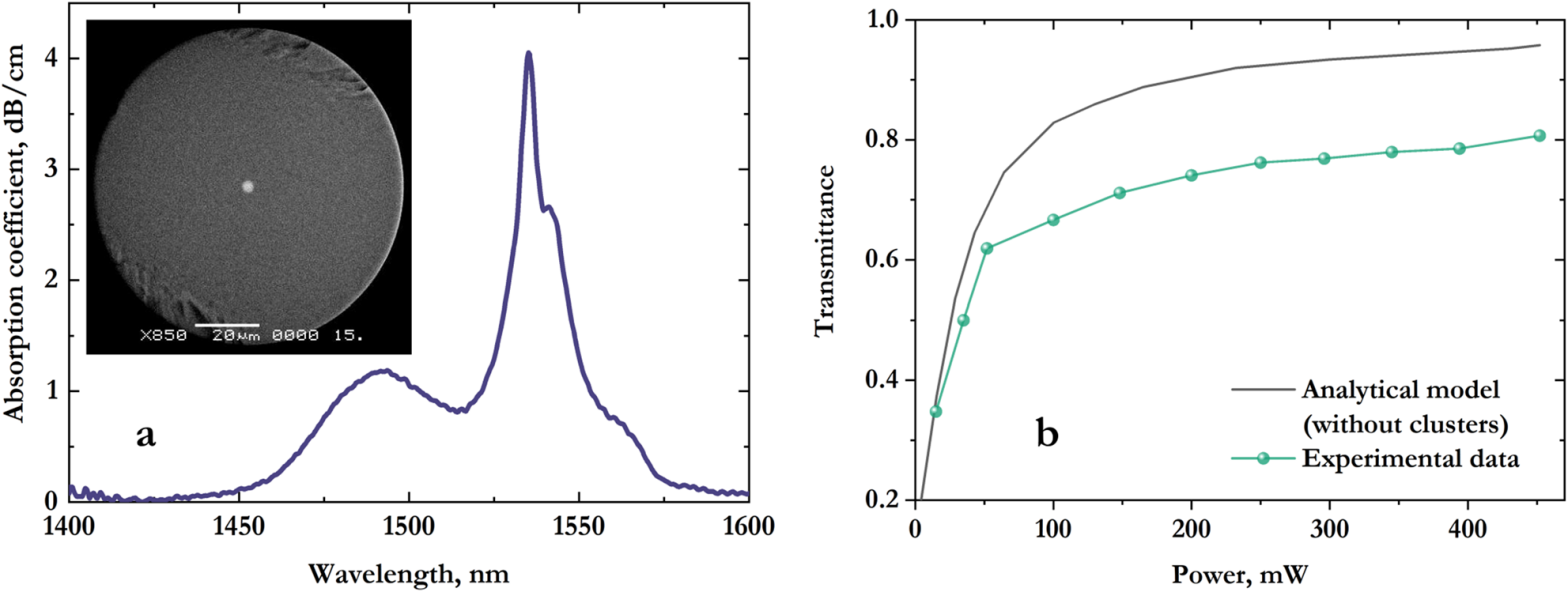
(**a**) Absorption spectrum of the fabricated composite fiber heavily doped with erbium ions. The inset shows cleaved fiber tip captured with electronic microscope. (**b**) Fiber transmittance as a function of pump power: experimental data (black) and analytical model (green).

Figure 1a shows the absorption spectra of the obtained fiber near the wavelength of 1,550 nm in the weak signal regime. The peak absorption is about 4 dB/cm at the wavelength of 1535 nm and about 1.4 dB/cm at a pump wavelength of 980 nm, which corresponds to the concentration of active ions *N* ≈ 1.6 × 10^20^ cm^−3^. The lifetime of Er^3+^ upper level ^4^I_13/2_ in the fiber was measured to be 3.5 ms. We believe that low radiation lifetime is caused by high concentration of OH^−^ groups in the initial phosphate glass.

It is known that optical fibers with a high concentration of active ions (*N* > 10^19^ cm^−3^) are subject to ion clustering, which in turn can lead to pulsed laser generation. In^17^, switching between pulsed and continuous wave lasing regimes depending on the concentration of clusters in the fiber and the pump power is obtained using numerical simulation. As a rule, in the case of a high-Q laser cavity, the cluster concentration should not exceed 10% to secure continuous generation near the threshold.

The following expression determines the fraction of ions that nonradiatively relax to the ground level^18^:

1 $$M = \, 10\log_{10} \left( {T_{m} /T_{e} } \right)/\alpha L$$

where *T*_*m*_ and *T*_*e*_ are the calculated and measured values of a fiber transmission at a pump wavelength, *α* is the absorption coefficient for a weak input signal, *L* is the fiber length. The transcendental equation is used to determine the value of *T*_*m*_^19^:

2 $$T_{m} {-} \, \exp [ - \alpha L + \left( {1 - T_{m} } \right)P_{in} /P_{is} ] = \, 0,$$

where *P*_*in*_ and *P*_*is*_ are the input power and the saturation power at the pump wavelength, respectively.

In order to determine the fraction of active ion clusters for the used active fiber, a transmittance at a pump wavelength of 980 nm was measured for a fiber section with a length of about 7 cm (Fig. 1b). The analytical model corresponds to the transmission of radiation at the pump wavelength in the absence of clusters (content 0%). Since our fiber is heavily doped with erbium ions, a certain fraction of ion clusters is present in its composition. The difference between the experimental data and the analytical model allows us to quantify this fraction in accordance with the approach described in^18^. Using Eq. (1), we made an estimate according to which the fraction of erbium ion clusters does not exceed 8%. For comparison, in the Er-80 fiber (3.7 × 10^19^ cm^−3^ doping concentration and 80 dB/m absorption at 1,530 nm)^20^ where the concentration of active ions is an order of magnitude lower, the percentage of clusters amounts to 14% that is almost 2 times higher than in our case. The difference is explained by the fact that the addition of impurities such as alumina^18^, helps to reduce clusters formation.

### DFB laser cavity written by femtosecond IR pulses

The main parameters that determine the minimum possible cavity length of a DFB laser are the gain of the active fiber *g*_*s*_, passive optical fiber loss *α*, and the mode coupling coefficient *κ* = *mπΔn/2n*_*eff*_Λ, where *m* is the FBG order, *Δn* is the amplitude of the refractive index modulation, *n*_*eff*_ is the effective refractive index of the core mode, Λ is the FBG period. The relation that determines the lasing threshold can be expressed as^3^:

3 $$g_{s} \approx \alpha + 4\kappa \exp ( - \kappa L),$$

In order to evaluate the gain, we used a 2-cm-long segment of an active fiber and a pump laser diode with a wavelength of 980 nm and an output power up to 600 mW. The measured gain coefficient at a wavelength of 1,550 nm for maximum pump power is *g*_*s*_ ≈ 2 dB/cm. The value of passive optical loss estimated from the transmission spectrum of a fiber waveguide in the wavelength region of 1,300 nm is *α* ≈ 4–5 dB/m. From Eq. (2), for a highly reflective FBG in the wavelength region of 1,550 nm at coupling coefficient κ = 450 m^−1^ and *g*_*s*_ = 2 dB/cm, the minimum DFB cavity length is estimated to be about 8 mm. For κ = 1,170 m^−1^ and *g*_*s*_ = 2 dB/cm the minimum cavity length amounts to about 4 mm.

Inscription of a FBG with a phase shift in the center of the structure was carried out by the femtosecond point-by-point method in the fiber workpiece, which was a section of an active fiber with a length of 8 mm to which passive tails of Nufern 1060-XP fiber were spliced. Femtosecond laser pulses (λ = 1,026 nm, Δ*t*_*p*_ = 232 fs) were focused into the core region of the active fiber using Mitutoyo 50 × Plan Apo NIR HR objective (NA = 0.6), and the fiber was moved using Aerotech ABL1000 high-precision air-bearing linear stage. A phase shift in the center of the FBG section was provided by amplitude modulation of 1 kHz sequence of femtosecond laser pulses, as proposed in^21^. The total length of the inscribed 2nd-order FBG is as short as 5.3 mm, and the resonance wavelength is about 1,550 nm. It should be noted that the advantages of the femtosecond pulse inscription technology were also used for fabricating DFB lasers in other unconventional active materials. In particular, the authors of^22^ demonstrated monolithic DFB laser based on waveguide-Bragg grating directly written an erbium- and ytterbium-doped phosphate glass with femtosecond pulses at 800 nm. The authors of^23^ demonstrated all-fiber DFB laser operating at 2.8 μm, where femtosecond pulses at 800 nm and phase-mask technique are used to inscribe π-phase-shifted FBG in a heavily erbium-doped fluoride fiber. In^24^ the authors propose a new technique for introducing phase shifts into the FBG structure during the writing of point-by-point FBGs with femtosecond laser pulses at 1,026 nm. In^25^ this technique was successfully applied for the realization of all-fiber DFB laser at 2.07 μm based on optical fiber with aluminosilicate glass core heavily doped with holmium ions.

**Figure 2:**
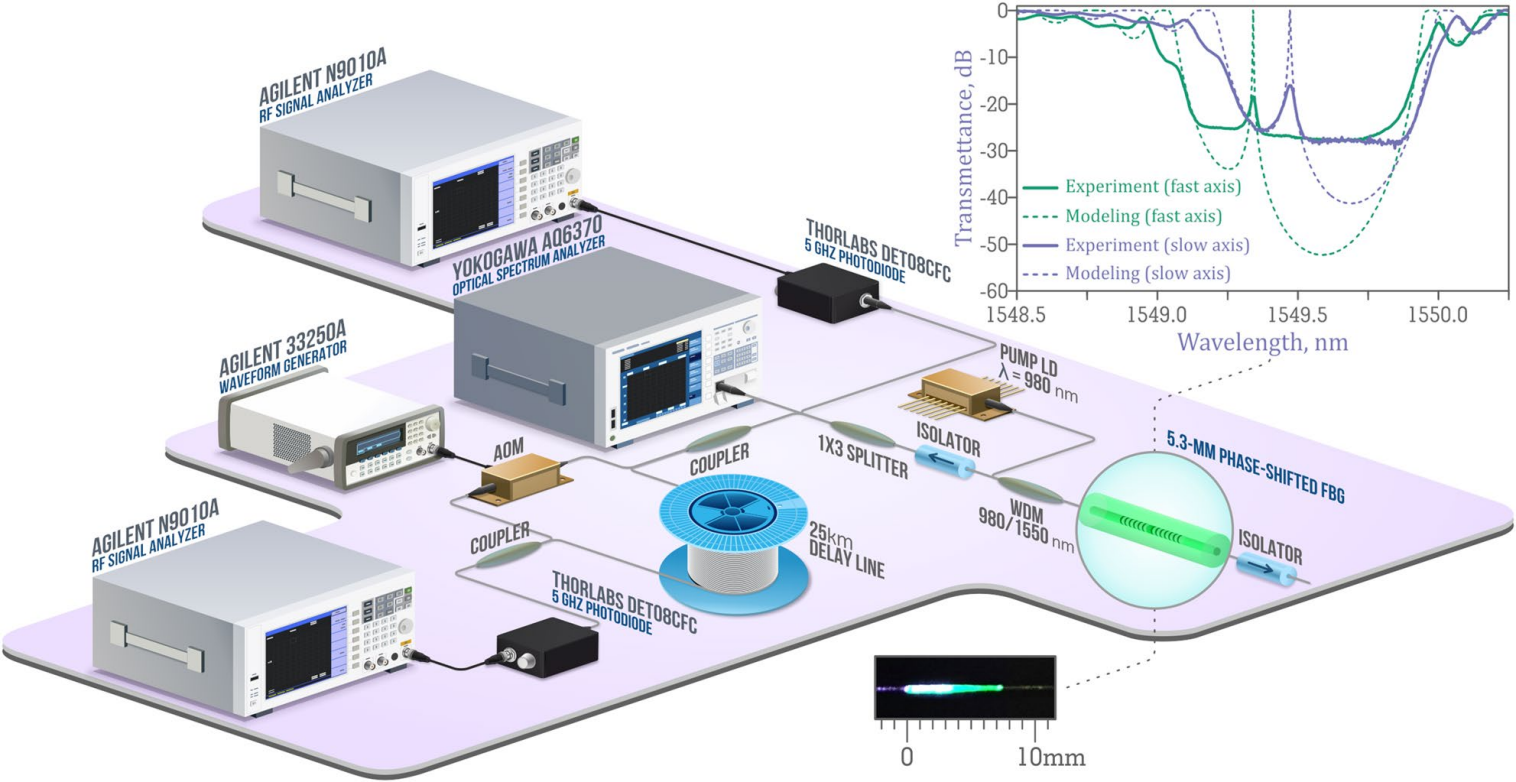
Scheme of the DFB laser and related measuring equipment. The inset at the top shows the transmission spectra of the FBG with a phase shift for fast and slow polarization axes; the inset at the bottom is a photograph of the DFB laser cavity while pumping.

The inset to Fig. 2 shows the transmission spectra of the inscribed phase-shifted FBG, which were measured for different polarization axes using an Apex AP2050 optical spectrum analyzer with a resolution of 1 pm, as well as the spectra obtained by numerical simulation. From their comparison one can see that the fast axis of the FBG has a higher coupling coefficient than the slow one, which is associated with the peculiarity of the method of femtosecond point-by-point inscription^26,27^. Numerical simulation with coupling coefficients *κ*_*fast*_ = 1,357 m^−1^ and *κ*_*slow*_ = 1,115 m^−1^ for the fast and slow axes respectively agrees well with the corresponding experimental spectra, despite the limited dynamic range at measuring the transmission spectra of the phase-shifted FBG. The simulation also allows us to estimate the phase shift value, which is ΔΛ = 0.37 μm (or 0.7π) for the FBG period of about Λ = 1.07 μm, and the birefringence value, which is *δn* = *Δn*_*fast*_* − Δn*_*slow*_* ≈ * 1.1 × 10^–4^ at the refractive index modulation Δ*n*_*fast*_* ≈ * 6.7 × 10^–4^.

The experimental scheme of the DFB laser based on the phase-shifted FBG inscribed in the active fiber is presented in Fig. 2. The DFB cavity is pumped with a single-mode laser diode with a wavelength of 980 nm and an output power up to 600 mW through the 980/1,550 nm wavelength division multiplexer (WDM). The edge of FBG coincides with the splicing point between the active fiber segment and passive fiber tail through which pump radiation is coupled. Thus, the part of active fiber that does not contain FBG did not affect the lasing threshold and other characteristics, since we measured the laser output radiation propagating in the opposite direction to the pump radiation.

To minimize the influence of back reflection from the components and fiber end facets, optical isolators were used on both sides of the DFB cavity. The laser output radiation counter propagating with the pump radiation was divided into 3 measuring channels by the 1 × 3 splitter. The measurements of relative intensity noise (RIN) were carried out using an Agilent N9010A radio frequency (RF) analyzer and a 5-GHz Thorlabs DET08CFC photodiode, the laser wavelength and output power were measured using a Yokogawa AQ6370 optical spectrum analyzer. We used a self-heterodyne technique based on the Mach–Zehnder interferometer (MZI) to measure the laser linewidth with a high precision^28^. One of MZI arms contains 25-km delay line corresponding to the limiting spectral resolution of ≈ 8.3 kHz. Another arm contains acousto-optic modulator (AOM) driven by Agilent 33250A waveform generator at a carrier frequency of 80 MHz, while RF signal is analyzed by Agilent N9010A/5-GHz Thorlabs DET08CFC set.

**Figure 3:**
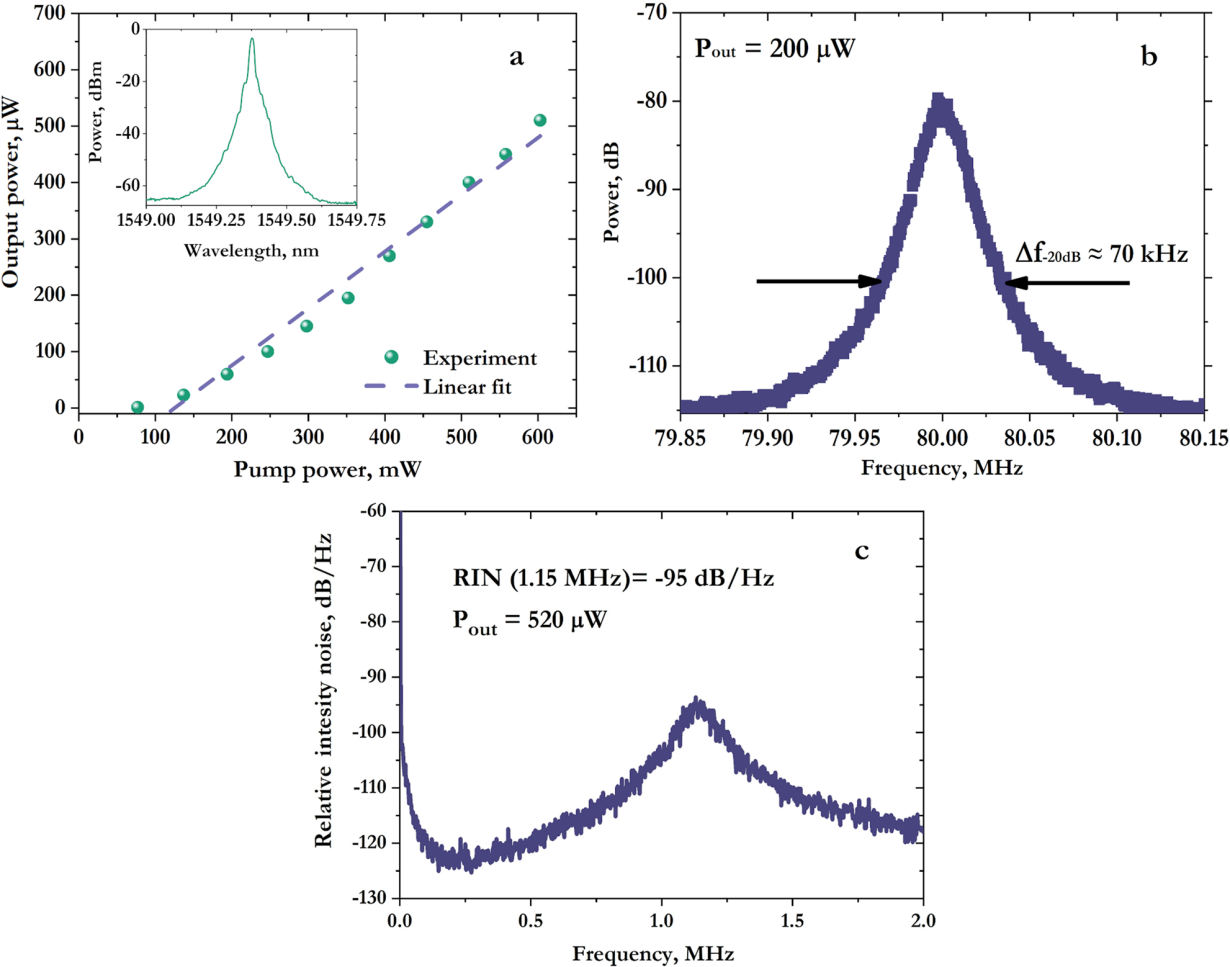
(**a**) The dependence of the output power on the pump power, the inset shows DBF laser spectrum at maximum output power. (**b**) RF spectrum of beat signals measured using self-heterodyne technique. The spectrum was averaged by 100 sweeps; the measurement time was 5 s. (**c**) Relative intensity noise measured at the maximum output power of the laser.

Figure 3a shows the dependence of the output radiation power on the pump power. The lasing threshold is observed at a pump power of ≈ 75 mW. We assume that the relatively high threshold power is due to the short ion lifetime at the metastable level τ ≈ 3.5 ms, since for a high-Q cavity *P*_*th*_ ~ 1/τ^3^. For comparison, in silicate glass Er^3+^ active fibers have τ ≈ 10 ms. The obtained differential efficiency (η ≈ 0.1%) is comparable with typical values for erbium DFB lasers, which have an order of magnitude longer cavity^29,30^. Lasing was observed only for the fast polarization axis at 1549.4 nm in a broad range of powers from the threshold to maximum power, see optical spectrum in the inset of Fig. 3a. This behavior is typical for resonators inscribed by femtosecond laser radiation and point-by-point writing technique^27^, where FBG’s coupling coefficient is different for different polarization axes. More precise self-heterodyne measurements give the value of beat-signal spectral width of about 70 kHz at a level of − 20 dB, which corresponds to the laser linewidth of 3.5 kHz (Fig. 3b).

Figure 3c shows the relative intensity noise (RIN) at the maximum laser power. For an erbium DFB laser, typical frequencies of the relaxation oscillations peak do not exceed 0.6 MHz ^3^, in our case this value is 1.15 MHz at an output power of 520 μW. This is because the frequency at which the RIN peak is located depends on the lifetime *τ* of active ions at a metastable level as well as the lifetime *τ*_*c*_ of the photon in the cavity as $\nu \sim 1/\sqrt{{\tau }{\tau }_{c}}$^31^. As mentioned earlier, the lifetime at the metastable level is almost 3 times less than that for standard commercially available fibers due to the presence of hydroxyl groups in the phosphate fiber. The measured level of RIN is − 95 dB/Hz at 1.15 MHz, which is a typical value for this type of laser without external active stabilization.

Thus, the power and spectral characteristics of the 5.3-mm DFB laser based on the heavily doped active fiber are similar to characteristics of DFB lasers with a cavity length of an order of magnitude greater. The influence of ion lifetime at a metastable level on the generation threshold and peak frequency RIN is also demonstrated. To the best of our knowledge, the DFB cavity length of 5.3 mm is the shortest reported for fiber lasers based only on erbium-doped active medium.

Despite the advantages of the femtosecond point-by-point writing method, fabrication of high-quality long FBGs is an issue due to errors occurring while positioning the modification region inside the fiber core, especially in the case of fibers with a relatively small mode field diameter. At the same time, inscription methods based on a phase mask overcome this limitation. Therefore, inscription of DFB laser cavity with a length > 10 mm was carried out using the phase mask and CW UV laser. Though photosensitivity of our active fiber is low, it is enough to fabricate relatively long gratings.

### DFB laser cavity written by UV radiation

To increase the photosensitivity of the active fiber, it was loaded with molecular hydrogen at atmospheric pressure of 100 bar for 10 days before the inscription. As a source of UV radiation, we used a CW frequency-doubled Ar-ion laser (λ = 244 nm) with the output power of ≈ 30 mW and the beam diameter of 1.3 mm. Periodic modulation of the refractive index in the core region was formed by the holographic inscription method^14^. The phase mask and active fiber were moved perpendicular to the propagation of UV radiation using a motorized linear translator. To form a phase shift in the center of the FBG structure the phase mask was shifted along the fiber using a piezoelectric element.

The lasing threshold was not reached for the DFB cavity length of 10 mm, which was formed by this technique. Numerical simulation gives the values Δ*n*_*fast*_ ≈ Δ*n*_*slow*_ ≈ 5 × 10^–5^ (*κ* = 101 m^−1^, *κL* = 4), which is an order of magnitude smaller than the similar values derived for the point-by-point phase-shifted FBG. One of the reasons for the small value of the induced Δ*n* is the reduced photosensitivity of the fiber due to the presence of phosphorus oxide in the fiber^10^. Therefore, the fabrication of high-Q FBG-based DFB cavities in such fibers is more complicated with CW UV radiation. For this reason, DFB cavity should be longer in order to reach lasing threshold. In^13^, where an optical fiber was manufactured using a similar technology, but with a lower content of erbium ions (1.1 wt%), a 50-mm DBR laser cavity was fabricated using pulsed UV radiation at 248 nm.

In the next experiment, we used a fiber workpiece with a 40-mm active fiber segment, in which a phase-shifted FBG was written over the entire length. Then, the inscribed FBG was placed in the experimental setup shown in Fig. 2 and similar measurements were made for this configuration.

**Figure 4:**
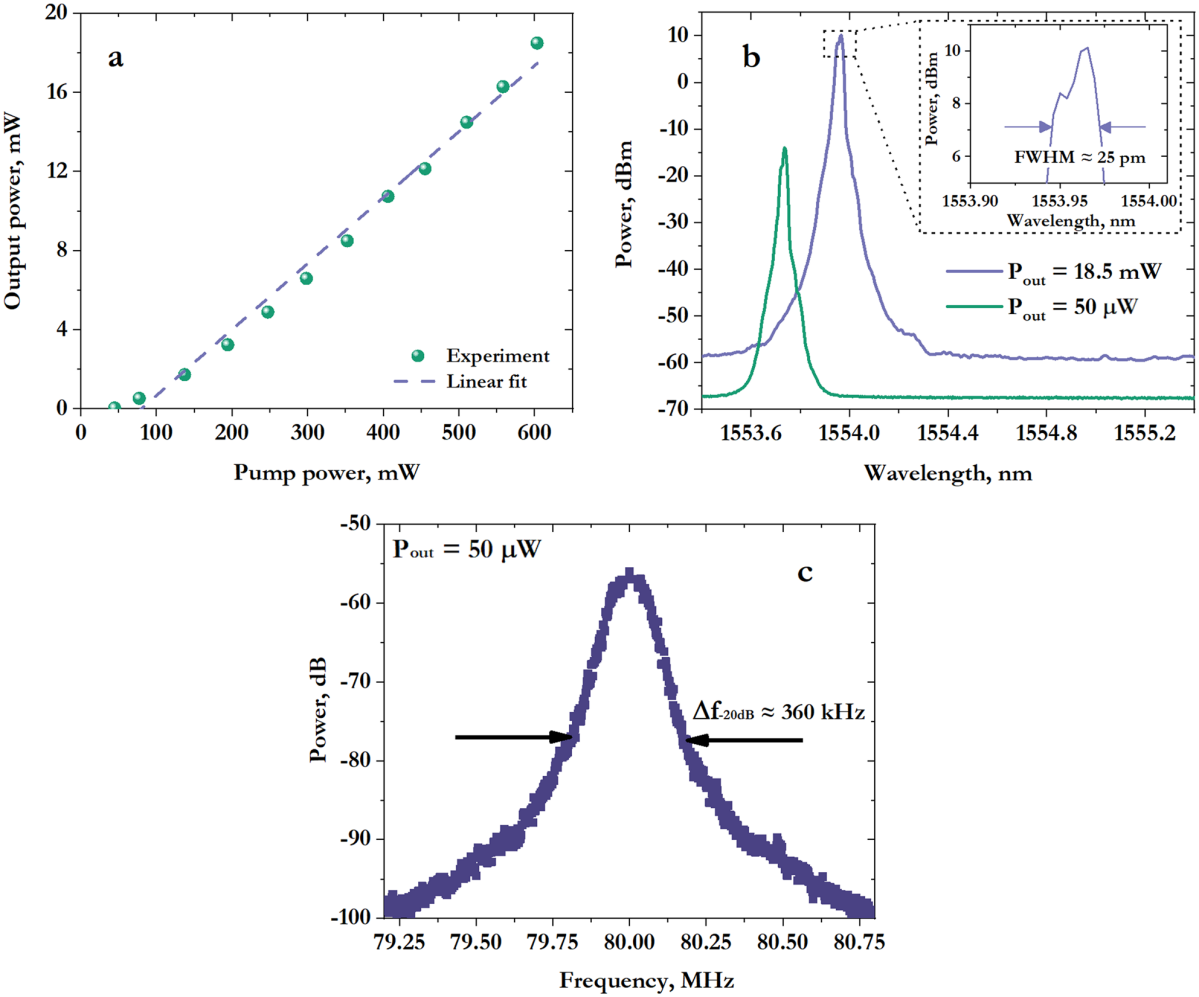
(**a**) The dependence of the output power on the pump power (**b**) Spectra of the DFB laser measured at different output powers (**c**) RF spectrum of two beat signals measured using self-heterodyne technique. The spectrum was averaged by 100 sweeps; the measurement time was 5 s.

Figure 4a shows the dependence of the laser output power on the pump power. The phase mask used for an FBG fabrication corresponds to a resonant wavelength of ≈ 1554 nm for the active fiber that we use. Even though the gain at this wavelength is less than 2 dB/cm, the output power of the DFB laser based on 40-mm phase-shifted UV FBG reached more than 18 mW at a pump power of about 600 mW. To the best our knowledge, the obtained efficiency η ≈ 3.3% is a record for Er^3+^-doped fiber DFB laser, since typical values are ~ 0.1%^29,30^.

Similar to the case of a DFB cavity fabricated with femtosecond radiation, we observed a single-frequency lasing regime near the threshold (*P*_*th*_ ≈ 45 mW) with a linewidth of 18 kHz (Fig. 4c). With further increase of the pump power, we observed an appearance of the second detuned lasing line, which we associate with the second orthogonal polarization mode with the approximately equal FBG’s strength *κ*_*fast*_*L ≈ κ*_*slow*_*L*. Lasing spectra at different output powers are provided in Fig. 4b.

Thus, a record efficiency *η* ≈ 3.3% for an erbium DFB laser has been achieved for 40-mm π-phase-shifted FBG which was fabricated with UV radiation. Single-polarization single-frequency lasing was observed only near the lasing threshold; achieving a single-polarization regime at higher power requires special selection techniques^32^. Moreover, we have found that the UV writing technique does not allow for the fabrication of short DFB resonators (< 10 mm) due to the limited magnitude of the induced Δ*n*. We associate this fact with a decreased photosensitivity of the fiber due to the high content of phosphorus oxide.

## Conclusion

Thus, the developed technology of phase shifted FBG inscription in specially designed Er^3+^-doped composite fiber by means of femtosecond pulses enables fabrication of the efficient DFB laser with cavity length of 5.3 mm that is the shortest reported for fiber lasers based only on Erbium-doped active medium, to the best of our knowledge. It has been shown that the power and spectral characteristics of such laser are comparable to those for conventional Er^3+^-doped fiber DFB lasers with much longer cavity. In the whole power range from the threshold to maximum power of ≈ 0.5 mW, a single-frequency generation with narrow linewidth (≈ 3.5 kHz) is observed in one linear polarization mode with peak relative intensity noise of − 95 dB/Hz at 1.15 MHz.

Using fabricated composite fiber we have also developed relatively long (≈ 40 mm) DFB laser by conventional FBG fabrication technology with CW UV laser and phase mask. The output power of such laser reaches more than 18 mW at a pump power of about 600 mW. So, the obtained efficiency η ≈ 3.3% is by an order of magnitude higher than typical efficiency of Er^3+^-doped fiber DFB laser being a record for this class of lasers, to the best our knowledge.

In practical sense, the developed techniques open a way to fabrication of Er^3+^-doped fiber DFB lasers operating in fiber transmission window around 1.55 µm with excellent output characteristics. Possibility of an FBG packaging in a desired footprint combined with low sensitivity to environment makes the technology attractive for sensing applications, in particular, in point-action active sensor arrays.
